# Zero superior vena cava injury lead extraction with rotational system: A contemporary experience

**DOI:** 10.1016/j.xjon.2024.11.010

**Published:** 2024-11-26

**Authors:** Iverson E. Williams, Omar M. Sharaf, Ryan Azarrafiy, Daniel Demos, Eric I. Jeng, Kirsten A. Freeman, John R. Spratt, Thomas M. Beaver

**Affiliations:** Division of Cardiovascular Surgery, Department of Surgery, University of Florida Health, Gainesville, Fla

**Keywords:** Evolution RL rotational sheath, lead extraction, superior vena cava injury

## Abstract

**Background:**

Transvenous cardiac implantable electronic device (CIED) lead extraction (TLE) is susceptible to superior vena cava (SVC) injury and can be performed in the operating room (OR) or electrophysiology lab via a mechanical device or laser-powered extraction. This study reflects a contemporary experience of mechanical right-left rotational extraction by cardiac surgeons in the OR.

**Methods:**

We conducted a retrospective single-center review of adult (age ≥18 years) TLE cases performed by cardiac surgeons between 2019 and 2021. Leads were extracted via a transvenous mechanical right-left controlled-rotation system in the OR under general anesthesia with transesophageal echocardiographic guidance. Procedural success was defined as complete extraction of all leads without major complications, based on the Heart Rhythm Society's 2017 guidelines.

**Results:**

A total of 210 leads were extracted from 104 patients, including 72 males (69%). The mean patient age was 63.8 ± 16.7 years, and 26 patients (25%) had undergone prior sternotomy. The most common indication for CIED extraction was infection (69%; n = 72). Removed CIEDs included single-chamber defibrillators (46%; n = 48), pacemakers (33%; n = 34), and cardiac resynchronization therapy devices (21%; n = 22). The mean age of the oldest extracted lead by patient was 9.79 ± 7.25 years. Procedural success was obtained in 95% of cases (99/104). The remaining cases included distal lead fracture (n = 3), inferior vena cava laceration necessitating sternotomy (n = 1), and tricuspid valve damage requiring delayed valve replacement (n = 1). There were zero SVC injuries, and procedure-related mortality was 0%.

**Conclusions:**

Mechanical, controlled-rotation TLE is effective and can be performed safely without SVC injury. TLE by cardiac surgeons in the OR enables rapid conversion to sternotomy in the event of major complications.

**Figure:**
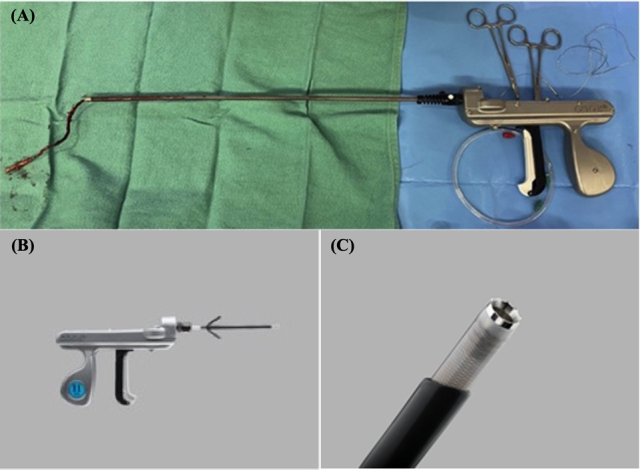
A, Evolution R/L Excluder following lead extraction. B, Evolution “Shorty” R/L excluder. C, The “Shorty” R/L excluder has a sharper blade at the tip for traversing through adhesions below the clavicle at the point of insertion.

**Central Message**

Performing transvenous lead extraction (TLE) with a mechanical rotational system is a safe maneuver with a low risk of complications. In the event of a complication, performing TLE in the operating room allows for rapid conversion to sternotomy.

**Perspective**

In this series, 104 patients underwent transvenous lead extraction (TLE) with the Evolution RL mechanical rotational system, in which 210 leads were extracted. Notably, superior vena cava injury, which is a devastating complication of TLE, and procedure-related mortality occurred in no cases. Despite the occurrence of major complications in 2 patients (1.9%), both patients survived following rapid conversion to sternotomy in the operating room.

Following an increase in the rate of cardiac implantable electronic device (CIED) lead implantations, the number of transvenous device lead extractions (TLEs) has increased as well.^1^^,^^2^ TLE is indicated in the setting of infection, malfunction, or upgrade of a preexisting cardiac device and poses challenges, as implanted leads can develop fibrous adhesions around adjacent structures, increasing the risk of procedural complications.^3^

One lethal complication of TLE requiring prompt management is superior vena cava (SVC) injury.^3^ A report from the Food and Drug Administration's MAUDE (Manufacturers and User Defined Experience) database revealed that most lead extractions resulting in injury to the SVC were associated with laser lead extraction.^4^ In a report from the Cleveland Clinic, out of 3528 lead extractions, there were 25 (0.8%) catastrophic complications, of which 23 (92%) involved a laser system that resulted in 16 SVC injuries.^5^

Extraction devices, such as mechanical and laser-powered devices, have improved the safety and efficacy of TLE.^6^ The Evolution RL (Cook Medical) is a bidirectional mechanical (right-left) rotational system. There is a paucity of literature regarding the outcomes of performing TLE in the operating room (OR) by surgeons with mechanical extraction devices versus in the electrophysiology lab with laser-powered devices. Importantly, performing TLE in the OR allows rapid conversion to sternotomy in the event of a complication.^3^ This study aimed to assess complete procedural success, complications, and 30-day mortality in adult patients (age ≥18 years) who underwent TLE in a cardiac surgery OR with the Evolution RL mechanical controlled-rotation system.

## Patients and Methods

### Study Population and Selection Criteria

We performed a retrospective review of adults (age ≥18 years) undergoing TLE with the Evolution RL mechanical controlled-rotation system by 4 cardiac surgeons at a single tertiary referral center between 2019 and 2021. Patients who underwent TLE with simple traction were excluded. University of Florida Institutional Review Board approval was obtained with a waiver of the need for consent (#IRB202100222). The data and findings of this study were conducted independently and are free of commercial bias. No funding or support was received from any commercial entity for the completion of this study.

### TLE Procedure

All cases were performed in a hybrid OR under general anesthesia with transesophageal echocardiography (TEE) guidance to assess for pericardial effusion and evaluate valve function. After standard prepping and draping, an 8 Fr common femoral venous sheath is placed for intravenous access to allow for large-volume resuscitation and deployment of a rescue balloon, femoral snaring of the lead, or temporary pacing. An incision is made over the subclavian pocket at the location of the previous incision, and the generator is exposed. Dissection is taken down to the left subclavian vein lead insertion point, deep to the pectoralis major muscle.

A 2-0 Vicryl purse-string suture is placed around the targeted lead and controlled with a Rummel tourniquet. For each lead, an inner stylet is used to facilitate a counterclockwise rotation to detach “screw-in” leads from the endocardium. A Liberator Beacon Tip locking stylet (Cook Medical) is then passed to the distal extent of the lead, aiding the creation of a rail for mechanical extraction. A mechanical right-left controlled-rotation system is passed over both the lead and locking stylet, depending on the size of the lead (9, 11, or 13 Fr). Under fluoroscopic guidance, the device is used to free surrounding intraluminal scar tissue, aiding lead extraction.

Following the removal of intended leads, the Vicryl suture is tied to secure hemostasis. The subclavian pocket is either closed or, in cases of severe infection, dressed with a vacuum sponge. The patient is typically monitored for 1 day postoperatively.^3^^,^^6^

### Primary and Secondary Outcomes

The primary outcome was the complete extraction of all leads without intraoperative SVC injury. Complications and procedural success were defined according to the Heart Rhythm Society's 2017 guidelines.^7^ Minor complications were undesired adverse events that required medical intervention but did not significantly affect patient function. Major complications were those that posed an immediate threat to life. Complete procedural success was defined as the complete extraction of all targeted leads without a major intraoperative complication.

### Statistical Analysis

Descriptive statistics were performed. Continuous variables are presented as mean ± SD or as median (interquartile range [IQR]), and categorical variables are presented as percentage (number). All analyses were performed using the SPSS version 28 (IBM).

## Results

### Study Population

During the study period, 104 patients underwent TLE with the Evolution RL mechanical controlled-rotation system. Patient baseline and demographic data are provided in Table 1. The cohort was 69.2% male (n = 72). The patients’ mean age at the time of surgery was 63.83 ± 16.74 years, mean body mass index was 30.01 ± 7.71, and mean left ventricular ejection fraction was 36.6 ± 17.1%. Common comorbidities included hypertension (77%; n = 80), congestive heart failure (68%; n = 71), diabetes (35%; n = 36), chronic kidney disease (23%; n = 24), nonischemic cardiomyopathy (33%; n = 34), and coronary artery disease (45%; n = 47). Prior sternotomy was present in 25% pf the patients (n = 26).

**Table 1:** Baseline data for all patients (N = 104)

Variable	Value
Male sex, n (%)	69.2 (72)
Age at time of surgery, y	
Mean ± SD	63.8 ± 16.7
Median (IQR)	66.5 (52.8-77.3)
BMI, kg/m^2^	
Mean ± SD	30.1 ± 7.71
Median (IQR)	28.9 (24.3-34.0)
Hypertension, n (%)	77 (80)
Diabetes, n (%)	35 (36)
Chronic kidney disease, n (%)	23 (24)
Coronary artery disease, n (%)	45 (47)
Congestive heart failure, n (%)	68 (71)
Nonischemic cardiomyopathy, n (%)	33 (34)
LVEF, %	
Mean ± SD	36.6 ± 17.1
Median (IQR)	35.0 (25.0-55.0)
Prior sternotomy, n (%)	25.0 (26)

*SD*, Standard deviation; *IQR*, interquartile range; *BMI*, body mass index; *LVEF*, left ventricular ejection fraction.

### Lead-Specific Data

Lead data are displayed in Table 2. A total of 210 leads were extracted. The most common indication was infection (69%; n = 72), followed by device malfunction (19%; n = 20), and need for device upgrade (6.7%; n = 7). In descending frequency, CIEDs extracted included implantable cardioverter-defibrillators (46.2%; n = 48), permanent pacemakers (32.7%; n = 34), and cardiac resynchronization therapy devices (21.2%; n = 22). The mean age of all leads extracted was 8.0 ± 5.74 years, with a median of 7.0 years (IQR, 4.0-11.0 years), while the mean age of the oldest extracted lead by patient was 9.79 ± 7.25 years. The heart chambers in which extracted leads were located included 81 right atrial leads (38.6%), 94 right ventricular leads (44.8%), and 18 coronary sinus leads (8.6%).

**Table 2:** Lead data

Variable	Value
Total number of leads extracted	210
Right atrium, % (n)	38.6 (81)
Right ventricle, % (n)	44.8 (94)
Coronary sinus, % (n)	8.6 (18)
Indication for extraction, % (n)	
Infection	69 (72)
Malfunction	19 (20)
Upgrade	6.7 (7)
Device type extracted, % (n)	
ICD	46.2 (48)
CRTD	21.2 (22)
PPM	32.7 (34)
Age of oldest lead, y	
Mean ± SD	9.79 ± 7.25
Median (IQR)	8.0 (4.3-14.0)
Age of extracted leads, y	
Mean ± SD	8.0 ± 5.74
Median (IQR)	7.0 (4.0-11.0)
Use of snare device, % (n)	1.9 (2)
New device placement, % (n)	44.5 (49)
ICD	55.1 (27)
LPM	22.4 (11)
PPM	22.4 (11)

*ICD*, Implantable cardioverter-defibrillator; *CRTD*, cardiac resynchronization therapeutic device; *PPM*, permanent pacemaker; *SD*, standard deviation; *IQR*, interquartile range; *LPM*, leadless pacemaker.

### Primary and Secondary Outcomes

Outcomes are outlined in Table 3. Complete procedural success was achieved in most cases (95%; n = 99). In 2 cases, TLE was initiated with the Evolution RL, but use of a snare device was required for full extraction. Following device explant, 49 patients (44.5%) required subsequent implantation of a new device, which included 27 (55.1%) implantable cardioverter-defibrillators, 11 (22.4%) leadless pacemakers, and 11 (22.4%) permanent pacemakers. Patients with lead infection who were pacemaker-dependent were treated with a screw-in electrode from the right internal jugular vein until later implantation.

**Table 3:** Outcome data (N = 104)

Variable	Value, % (n)
Complete procedural success	95.0 (99)
Major complications	1.9 (2)
Minor complications	3.9 (4)
Thirty-day mortality	1.9 (2)

Complications that prevented complete procedural success included 3 distal lead fractures and 2 major complications. For the 3 leads that were fractured during extraction, the time from implantation to extraction attempt ranged from 2.5 to 10.5 years.

One of the major complications was an inferior vena cava (IVC) injury, which required an emergent sternotomy with IVC repair using a bovine pericardial patch. This patient had no significant past medical history, including no prior sternotomy. The other major complication was a tricuspid valve injury, which occurred during retraction of a ventricular lead traversing the valve. This patient eventually required an open tricuspid valve replacement. To avoid this injury, bringing the evolution sheath down below the tricuspid valve during extraction is recommended to. Similar to the patient with the IVC injury, this patient also did not have a history of prior sternotomy. The age of the leads extracted was 7 years for the IVC injury and 5 years for the tricuspid valve injury. Ultimately, both patients survived and were discharged in stable condition. Secondary outcomes were 4 (3.9%) minor intraoperative complications, including 3 pocket hematomas and 1 superficial wound infection. There were 2 (1.9%) 30-day mortalities, in which both patients presented with staphylococcal infections and later decompensated to septic shock postoperatively. There were no cases of intraoperative SVC injury or procedure-related mortality.

## Discussion

Cardiac lead extractions are becoming increasingly common with the expanding use of CIED lead implantations.^2^ Mechanical extraction and laser-powered extraction are the 2 primary approaches for TLE, which can be performed in an OR or an electrophysiology lab.^3^ However, TLEs are susceptible to intraoperative complications, including SVC injury, which has spurred debate surrounding the efficacy of TLE approaches and the safest setting for TLE.^4^^,^^5^

In this study, 210 leads were mechanically extracted from 104 patients by cardiac surgeons in the OR with 95% (n = 99) complete procedural success, no SVC injuries, and no procedure-related mortality. Starck and colleagues^8^ also evaluated complete procedural success in TLEs performed with the Evolution RL mechanical controlled-rotation system. In their cohort of 40 patients, 46 of 52 leads (88.5%) were extracted, with complete procedural success in 34 patients (85%). In the remaining 6 patients, there were 5 partial extractions and 1 failed extraction. Notably, the authors observed no major intraoperative complications.

Although rare, devastating intraoperative complications can occur with TLE, including SVC injury, which can lead to death without prompt intervention. In 2014, Brunner and colleagues^5^ reported that 25 of 3258 TLE patients (0.8%) had catastrophic complications necessitating emergent surgical or endovascular intervention. Laser extraction was used in 23 of these 25 patients (92%) with 16 SVC injuries (64%), suggesting a possible correlation between SVC injury and laser-powered devices. Hauser and colleagues^4^ reviewed the MAUDE database for TLEs performed between 1995 and 2008. Cardiovascular events occurred during 105 lead extractions, with 57 deaths (54.3%). Twenty-five deaths (43.9%) occurred when a laser system was used, and 6 (10.5%) occurred during mechanical extractions. Thirty-four vascular injuries occurred with the laser system, compared to only 2 vascular injuries with the Evolution RL, highlighting the risks of laser versus mechanical extractions.

Bracke and colleagues^9^ performed a single-center analysis of the Evolution RL device with 250 leads. Major complications occurred with 3 leads (1.2%), including 1 SVC injury. Sharma and colleagues^3^ similarly performed a single-center study assessing the safety and efficacy of performing TLE with the Evolution RL. In their cohort, 683 leads were extracted from 400 patients, with complete lead removal and clinical success rates of 97% and 99.75%, respectively. Major complications occurred in 6 patients (1.5%), with no SVC injuries or deaths. Later, Sharma and colleagues^10^ performed a multicenter study evaluating mechanical TLEs and found complete procedural success in 443 of 460 leads extracted (96.3%), major complications in 6 of 230 patients (2.6%), and no isolated SVC injuries or mortalities. As with the prior experiences, the current study performed by surgeons in the operating room adds to the literature supporting that the mechanical controlled-rotational system can produce excellent results with minimal risk of vascular injury and procedure-related mortality.

### Limitations

This study is a retrospective analysis that lacks an experimental design, making it vulnerable to biases. Another limitation involves the absence of a comparison group. As this study prioritized the experience of 4 cardiac surgeons using the Evolution RL, patients who underwent TLE with simple traction were excluded. In addition, laser-powered extractions at our center are not performed by the cardiac surgery team. Finally, the final analysis included a small sample size, because TLE cases in which the Evolution RL was not used were excluded.

## Conclusions

The Evolution RL mechanical controlled-rotational system is a safe and effective method for TLE, with low complication and mortality rates. Mechanical TLE by cardiac surgeons in hybrid ORs under TEE guidance is ideal, with an additional safety level, as it allows for immediate conversion to sternotomy. Importantly, cardiac surgeons should consider adding mechanical TLE to their skill set, which will help make future lead extractions safer for patients.

## Conflict of Interest Statement

The authors reported no conflicts of interest.

The *Journal* policy requires editors and reviewers to disclose conflicts of interest and to decline handling or reviewing manuscripts for which they may have a conflict of interest. The editors and reviewers of this article have no conflicts of interest.
